# Novel Ag-Bridged Z-Scheme CdS/Ag/Bi_2_WO_6_ Heterojunction: Excellent Photocatalytic Performance and Insight into the Underlying Mechanism

**DOI:** 10.3390/nano14030315

**Published:** 2024-02-04

**Authors:** Fangzhi Wang, Lihua Jiang, Guizhai Zhang, Zixian Ye, Qiuyue He, Jing Li, Peng Li, Yan Chen, Xiaoyan Zhou, Ran Shang

**Affiliations:** School of Resources and Environmental Engineering, Shandong Agriculture and Engineering University, Jinan 250100, China; jiangli8227@sina.com (L.J.); zgzok2005@163.com (G.Z.); 18511259982@163.com (Z.Y.); ajheqiuyue@163.com (Q.H.); ripplelj@126.com (J.L.); lipengjenny@126.com (P.L.); ychen0612@163.com (Y.C.); yaling110@163.com (X.Z.); sr1982@sdaeu.edu.cn (R.S.)

**Keywords:** Z-scheme, photocatalysis, CdS/Ag/Bi_2_WO_6_, Ag-bridged, visible light

## Abstract

The construction of semiconductor heterojunction photocatalysts that improve the separation and transfer of photoinduced charge carriers is an effective and widely employed strategy to boost photocatalytic performance. Herein, we have successfully constructed a CdS/Ag/Bi_2_WO_6_ Z-scheme heterojunction with an Ag-bridge as an effective charge transfer channel by a facile process. The heterostructure consists of both CdS and Ag nanoparticles anchored on the surface of Bi_2_WO_6_ nanosheets. The photocatalytic efficiency of the CdS/Ag/Bi_2_WO_6_ system was studied by the decontamination of tetracycline (TC) and Rhodamine B (RhB) under visible light irradiation (λ ≥ 420). The results exhibited that CdS/Ag/Bi_2_WO_6_ shows markedly higher photocatalytic performance than that of CdS, Bi_2_WO_6_, Ag/Bi_2_WO_6_, and CdS/Bi_2_WO_6_. The trapping experiment results verified that the ^•^O_2_^−^ and h^+^ radicals are the key active species. The results of photoluminescence spectral analysis and photocurrent responses indicated that the CdS/Ag/Bi_2_WO_6_ heterojunctions exhibit exceptional efficiency in separating and transferring photoinduced electron−hole pairs. Based on a series of characterization results, the boosted photocatalytic activity of the CdS/Ag/Bi_2_WO_6_ system is mostly due to the successful formation of the Ag-bridged Z-scheme heterojunction; these can not only inhibit the recombination rate of photoinduced charge carriers but also possess a splendid redox capacity. The work provides a way for designing a Z-scheme photocatalytic system based on Ag-bridged for boosting photocatalytic performance.

## 1. Introduction

The rapid advancement of human society has led to an increasing focus on the issues of energy scarcity and environmental pollution. As a promising and eco-friendly technology that uses solar energy to address environmental problems and energy scarcity, semiconductor-based photocatalysis has recently received significant attention [1,2,3,4]. However, the traditional photocatalysts are only photoexcited in the ultraviolet, which only accounts for 4% of the total solar spectrum, thus considerably restraining their practical application [5,6]. Therefore, it is imperative to develop new visible-light-driven (VLD) photocatalysts [7].

Bismuth tungstate (Bi_2_WO_6_), a representative aurivillius oxide, possesses a unique layered structure composed of alternating [WO_4_]^2−^ octahedral layers and [Bi_2_O_2_]^2+^ layers; this was advantageous for the transmission of photogenerated carriers. Bi_2_WO_6_ has a suitable band gap of approximately 2.7 eV, which has been regarded as a promising VLD photocatalyst [8,9]. In addition, Bi_2_WO_6_ possesses numerous advantages, such as chemical stability, nontoxicity, and corrosion resistance. However, the UV-to-visible photo-absorption region of Bi_2_WO_6_ is shorter than approximately 450 nm. And swift recombination of photoinduced charge carriers extremely restricts its energy conversion efficiency [10,11]. To surmount these problems and improve the photocatalytic properties of pristine Bi_2_WO_6_, various techniques have been developed, such as morphological control [12,13], noble metal element doping [14,15], non-noble metal element doping [16,17], building heterojunction nanocomposite [18,19], and so on. Among these, the construction of heterostructures is a promising method, particularly the construction of Z-scheme photocatalytic systems [20,21]. The Z-scheme photocatalytic system can not only improve the transfer efficiency of photoexcited electrons and holes but also ensure a powerful redox capacity [22]. 

Among the connection modes of Z-scheme photocatalytic systems, ternary semiconductor/conductor/semiconductor Z-scheme heterojunctions in which two different semiconductors have a matching band structure can prominently enhance the photocatalytic performance [23,24]. Recently, noble metals (such as Ag, Pt, and Au) have been used as charge-carrying mediators, which can quickly transfer interfacial charge between two semiconductors. Besides, noble metal nanoparticles have a surface plasmon resonance during photocatalytic reactions. In recent years, various semiconductor/noble-metal/semiconductor Z-scheme heterojunctions, such as CdS/Ag/g-C_3_N_4_ [25], g-C_3_N_4_@Ag/BiVO_4_ [26], Co_3_O_4_/Ag/Bi_2_WO_6_ [27], CdS/Au/BiVO_4_ [28], BaTiO_3_/Au/g-C_3_N_4_ [29], g-C_3_N_4_/Ag/MoS_2_ [30], g-C_3_N_4_/Pt/Bi_2_WO_6_ [31], BiVO_4_/Au/CdS [32], MoS_2_/Au/g-C_3_N_4_ [33], etc., have been successfully synthesized. Xiao et al. [34] constructed C_3_N_4_@Ag-Bi_2_WO_6_ by a facile process, and the ternary system showed a boosted photocatalytic capacity for degrading RhB and producing H_2_ than that of single- and two-component systems; this was mainly due to the Z-scheme delivery mechanism. Gao et al. [35] successfully prepared BiVO_4_/Ag/CdS Z-scheme heterojunction, which attained improved ability in synergistic adsorption and photocatalytic degradation of fluoroquinolones. Hence, constructing a ternary semiconductor/noble metal/Bi_2_WO_6_ Z-scheme heterojunction could be a very promising strategy to obtain excellent photocatalytic activity. 

Cadmium sulfide (CdS) is a narrow-bandgap semiconductor with a band gap of about 2.4 eV, which has been attracting much attention for environmental contaminant purification and hydrogen generation [36,37,38]. As a consequence, CdS is usually coupled with various photocatalysts to enhance visible light absorption performance and the separation ability of photoinduced charge carriers [39,40,41]. Besides, CdS is a very suitable semiconductor for assembling heterojunctions based on Bi_2_WO_6_, because the CB and VB of CdS are matched well with Bi_2_WO_6_ [42,43,44]. Zhang et al. [45] prepared Z-scheme CdS/Bi_2_WO_6_ heterojunction via a simple hydrothermal method, and they found that 15% CdS/Bi_2_WO_6_ photocatalysts could remove 60.82% of Cr(VI) and photodegrade almost all Rhodamine B within 1 h.

In this study, we design and synthesize ternary CdS/Ag/Bi_2_WO_6_ Z-scheme heterojunction by a facile process. The photocatalytic experiments exhibited that this ternary CdS/Ag/Bi_2_WO_6_ Z-scheme displayed excellent photocatalytic performances toward photodegrading Rhodamine B (RhB) and tetracycline (TC) under visible-light irradiation. The Ag nanoparticles can act as a charge transfer bridge between Bi_2_WO_6_ and CdS; this could boost the transfer rate of photoinduced electrons and holes in this Z-scheme system. Moreover, a plausible mechanism was investigated and proposed for explaining the excellent photocatalytic performance of CdS/Ag/Bi_2_WO_6_ heterojunctions.

## 2. Materials and Methods

### 2.1. Preparation of Bi_2_WO_6_

Bi_2_WO_6_ photocatalysts were synthesized via a simple hydrothermal method. Dissolve 2 mmol Bi(NO_3_)_3_·5H_2_O, 1 mmol Na_2_WO_4_·2H_2_O, and 0.05 g of cetyltrimethylammonium bromide (CTAB) in diluted nitric acid and vigorously stir for 30 min to acquire a uniform suspension. The pH of the aforementioned suspension was adjusted to approximately 7 by NaOH solutions. After stirring for 30 min, the resultant solution was poured into a 100 mL Teflon-lined stainless autoclave and heated at 180 °C for 24 h. The precipitate was subsequently filtered and washed with distilled water and ethanol several times, then dried at 80 °C for 8 h.

### 2.2. Preparation of Ag/Bi_2_WO_6_

The Ag/Bi_2_WO_6_ photocatalyst was prepared using a photo-reduction method. To be specific, a certain amount of AgNO_3_ (0.05 mmol) was added to 50 mL of distilled water and then stirred until AgNO_3_ was completely dissolved in the dark. Afterward, the as-synthetized Bi_2_WO_6_ (1 mmol) was added to the AgNO_3_ solution and irradiated by 500 W Xe light (1 h) with vigorous stirring. Then the Ag/Bi_2_WO_6_ was collected and dried in a vacuum oven at 60 °C for 8 h. 

### 2.3. Preparation of CdS/Ag/Bi_2_WO_6_

The CdS/Ag/Bi_2_WO_6_ was prepared using a precipitation method. A certain amount of Cd(NO_3_)_2_·4H_2_O and 0.2 g of Ag/Bi_2_WO_6_ were dispersed in 30 mL of distilled water with ultrasonic vibration for 30 min. Subsequently, 20 mL of Na_2_S solution was dropwise added to the above solution and stirred for 4 h. The precipitate was filtered and washed with distilled water and ethanol several times, and finally dried at 80 °C overnight. The mass ratio of CdS:Ag/Bi_2_WO_6_ was controlled to be 0.04. Similarly, CdS/Bi_2_WO_6_ were prepared under the same conditions.

### 2.4. Characterization of Photocatalysts

The phase structures of the prepared samples were measured by X-ray diffraction (XRD) (D/MAX-RB; Rigaku, Tokyo, Japan). The diffraction patterns were examined in the 2θ range from 20° to 80° with a Cu Kα source (λ = 0.15405) running at 40 kV and 30 mA. The morphology of the samples was examined by scanning electron microscopy (SEM; S-4800; Hitachi, Hitachi-shi, Japan). Transmission electron microscopy (TEM) and high-resolution TEM (HRTEM) images were analyzed with a transmission electron microscope (FEI Talos F200X G2, Thermo Scientific, Waltham, MA, USA) at an accelerating voltage of 200 kV. The UV–vis diffuse reflectance spectra (DRS) were collected via a UV–vis spectrophotometer (T9s; Persee, Beijing, China) equipped with an integrating sphere. Barium sulfate (BaSO_4_) was used as the reference. Photoluminescence (PL) spectra data of the samples were recorded by a fluorescence spectrophotometer (F-4500; Hitachi, Japan).

### 2.5. Photocatalytic Experiments

The photocatalytic performances of the photocatalysts were assessed via the photodegradation of Rhodamine B (RhB) and tetracycline (TC) under visible light irradiation. A 400 W Xe lamp with a cut-off filter λ ≥ 420 nm served as a light source. In each photocatalytic degradation test, 40 mg of the as-obtained photocatalyst was dispersed in 40 mL of 10 mg/L RhB solution (or 20 mg/L TC). Prior to light irradiation, the prepared suspensions were stirred in the dark for 1 h to reach adsorption-desorption equilibrium. The RhB and TC concentrations were recorded by a UV–vis spectrophotometer at 553 and 357 nm, respectively. The degradation performance was evaluated using the ratios (C/C_0_) of the RhB and TC concentrations (C_0_ was the initial concentration, and C was the concentration at a given time).

### 2.6. Photoelectrochemical Measurements

Photoelectrochemical measurements were performed on an electrochemical workstation (5060F; RST, Zhengzhou, China) in a conventional three-electrode system with a 0.5 M Na_2_SO_4_ aqueous solution. The samples, a saturated calomel electrode, and a Pt wire were used as the working, reference electrode, and counter electrodes, respectively. The light source was provided by a 100 W incandescent lamp with a 420 nm cut-off filter. The working electrode was manufactured as follows: 5 mg of photocatalyst was dispersed homogeneously in a certain amount of Nafion solution and ethanol (*v*/*v* = 30:1). Finally, the as-prepared samples were loaded onto the bottom middle of ITO glass with a diameter of 6 mm. Then the photocurrents of the samples with the light on and off were measured at 0.8 V. 

## 3. Results and Discussion

### 3.1. Crystal Structure Analysis

**Figure 1:**
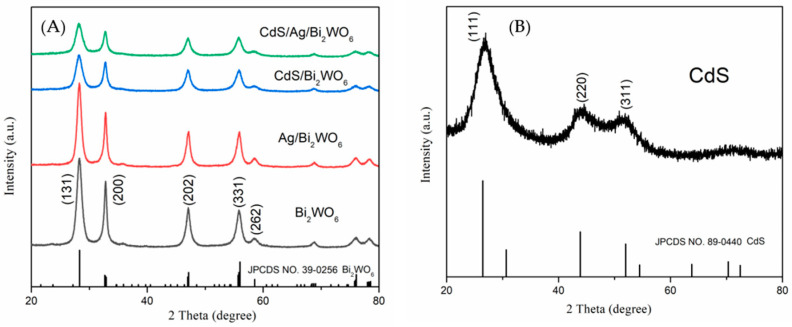
XRD patterns of Bi_2_WO_6_, Ag/Bi_2_WO_6_, CdS/Bi_2_WO_6_, CdS/Ag/Bi_2_WO_6_ (**A**), and CdS (**B**).

The crystal structures of Bi_2_WO_6_, Ag/Bi_2_WO_6_, CdS/Bi_2_WO_6_, CdS/Ag/Bi_2_WO_6_, and CdS were investigated using XRD. From Figure 1A, all diffraction peaks were completely corresponding to the structure of orthorhombic Bi_2_WO_6_ (JCPDS Card No. 39-0256). The characteristic peaks at 2θ = 28.3°, 32.8°, 47.1°, 55.8°, and 58.5° were attributed to the (1 3 1), (2 0 0), (2 0 2), (3 3 1), and (2 6 2) crystal planes, respectively. Comparing the curves of Bi_2_WO_6_ and Ag/Bi_2_WO_6_, it can be observed that they have similar patterns. This finding was consistent with the previous results [34]. This may be because of the low loading amount of Ag nanoparticles in the heterojunction. Furthermore, no characteristic diffraction peaks for CdS were observed in CdS/Bi_2_WO_6_ and CdS/Ag/Bi_2_WO_6_, which could be caused by the high dispersion, small particles, and small amount of CdS dopant. Similar results were found in CdS/Bi_2_WO_6_ [42] and CdS/BiOCl [46]. Figure 1B displays the XRD pattern of pure CdS, which can be assigned to the cubic phase of CdS (JCPDS Card No. 89-0440) [47]. The diffraction peaks at 26.4°, 43.9°, and 51.9° were well-matched with the crystal planes of (1 1 1), (2 2 0), and (3 1 1) of CdS, respectively. The existence of Ag nanoparticles and/or CdS in CdS/Ag/Bi_2_WO_6_ composites was further identified by TEM analysis. 

### 3.2. Morphology Characterization

**Figure 2:**
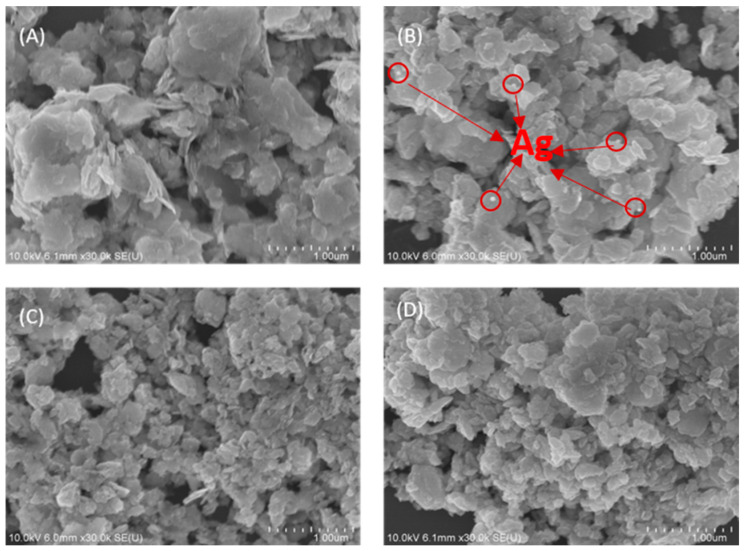
SEM images of (**A**) Bi_2_WO_6_, (**B**) Ag/Bi_2_WO_6_, (**C**) CdS/Bi_2_WO_6_, and (**D**) CdS/Ag/Bi_2_WO_6_.

The morphologies of Bi_2_WO_6_, Ag/Bi_2_WO_6_, CdS/Bi_2_WO_6_, and CdS/Ag/Bi_2_WO_6_ composites were studied by scanning electron microscope (SEM; S-4800; Hitachi, Hitachi-shi, Japan). As could be observed from Figure 2A, the Bi_2_WO_6_ showed an aggregated nanosheet-like microstructure. It was worth noting that Ag nanoparticles on the surface of Bi_2_WO_6_ were found, indicating that Ag nanoparticles were successfully deposited on the Bi_2_WO_6_ surface (Figure 2B). The SEM images of CdS/Bi_2_WO_6_ and CdS/Ag/Bi_2_WO_6_ composites (Figure 2C,D) were found to be similar to pure Bi_2_WO_6_. This similarity could be attributed to the use of the same original Bi_2_WO_6_ material, as well as high dispersion and the small particle size of CdS in the composites.

**Figure 3:**
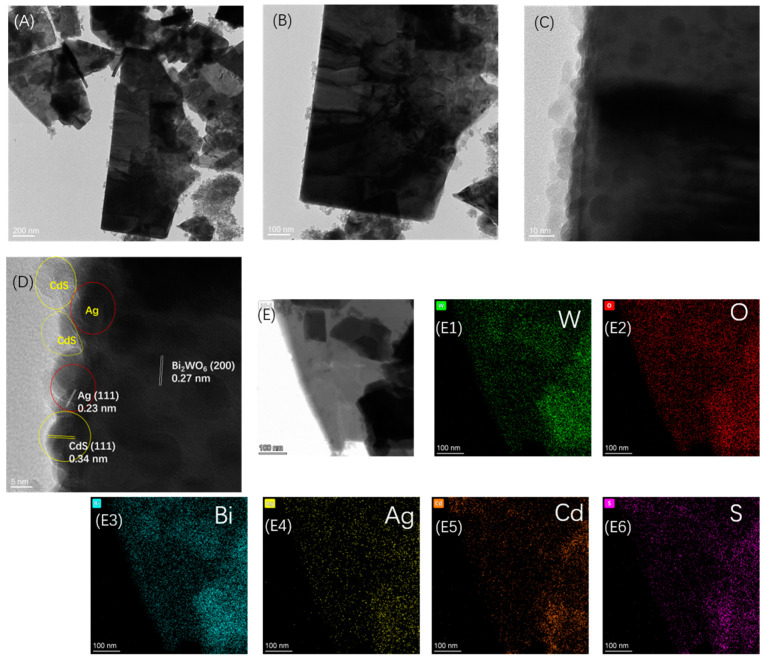
TEM images (**A**,**B**) and HRTEM images (**C**,**D**) of CdS/Ag/Bi_2_WO_6_; (**E**,**E1**–**E6**) the elemental mapping of W, O, Bi, Ag, Cd, and S of CdS/Ag/Bi_2_WO_6._

The morphologies of the CdS/Ag/Bi_2_WO_6_ were further observed by transmission electron microscopy (TEM) and high-resolution transmission electron microscopy (HRTEM). From Figure 3A,B, the CdS/Ag/Bi_2_WO_6_ sample showed an irregular and nanosheet-like microstructure. The HRTEM image (Figure 3C) displays that some nanoparticles have grown on the Bi_2_WO_6_ nanosheet. It is worth noting that the edges of Bi_2_WO_6_ nanosheets appear to have two different types of nanoparticles, which may be Ag and CdS nanoparticles. The HRTEM image (Figure 3D) shows the crystal plane spacing of 0.23, 0.34, and 0.27 nm; these correspond to the (1 1 1) plane of Ag nanoparticles, the (1 1 1) plane of CdS, and the (2 0 0) plane of Bi_2_WO_6_, respectively. Furthermore, Ag nanoparticles loaded on Bi_2_WO_6_ nanosheets are in close contact with CdS. It shows that CdS and Ag nanoparticles were successfully supported on the Bi_2_WO_6_ nanosheet, which could be beneficial for charge separation within the Z-scheme CdS/Ag/Bi_2_WO_6_ heterojunctions in comparison to pure Bi_2_WO_6_. Moreover, the elemental mapping method was employed to investigate the composition distribution in CdS/Ag/Bi_2_WO_6_ samples. As depicted in Figure 3E1–E6, W, O, Bi, Ag, Cd, and S elements are evenly distributed throughout the CdS/Ag/Bi_2_WO_6_ sample. And the element mapping distribution of Ag, Cd, and S proved evidence that CdS, Ag, and Bi_2_WO_6_ are closely combined. Combined with the TEM results, it can be indicated that CdS and Ag nanoparticles were evenly loaded on the surface of Bi_2_WO_6_ nanosheets. 

### 3.3. Optical Properties

**Figure 4:**
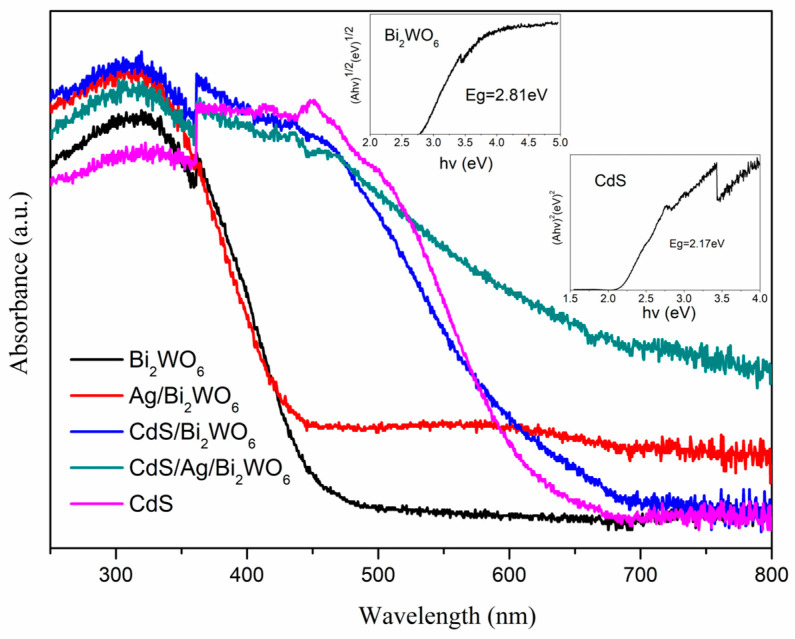
DRS spectra of pure Bi_2_WO_6_, Ag/Bi_2_WO_6_, CdS/Bi_2_WO_6_, and CdS/Ag/Bi_2_WO_6_; the inset shows the band gap energies of CdS and Bi_2_WO_6_.

The light absorption properties of the obtained samples were characterized by UV–vis DRS, as illustrated in Figure 4. The absorption edges were observed at approximately 450 nm and 650 nm for Bi_2_WO_6_ and CdS, respectively. The optical absorption edge of the CdS/Bi_2_WO_6_ composite was distinctly red-shifted compared with Bi_2_WO_6_, which could be assigned to the forming heterojunction between CdS and Bi_2_WO_6_. Meanwhile, after Ag nanoparticles growth on the surface of Bi_2_WO_6_ nanosheets, Ag/Bi_2_WO_6_ had a wide absorption in the visible light region; this may be attributed to the surface plasmon resonance effect of spatially confined electrons in Ag nanoparticles [48]. Compared with all other samples, the obtained CdS/Ag/Bi_2_WO_6_ ternary system exhibited enhanced visible light absorption, which may be on account of the synergetic effect of CdS and Ag. These results reveal that the as-prepared CdS/Ag/Bi_2_WO_6_ heterojunction photocatalyst had an excellent visible-light absorption range and thus produced more photoinduced electron−hole pairs, as demonstrated subsequently. The Kubelka-Munk formula: *ahv = A(hv − Eg)^n/2^* was used to estimate the band gap values of CdS and Bi_2_WO_6_ [49]. For CdS and Bi_2_WO_6_, the values of n are 1 and 4, respectively [50,51]. From the inset of Figure 4, the band gap values of CdS and Bi_2_WO_6_ are approximately 2.17 eV and 2.81 eV, respectively.

### 3.4. Photocatalytic Performances of the Samples

**Figure 5:**
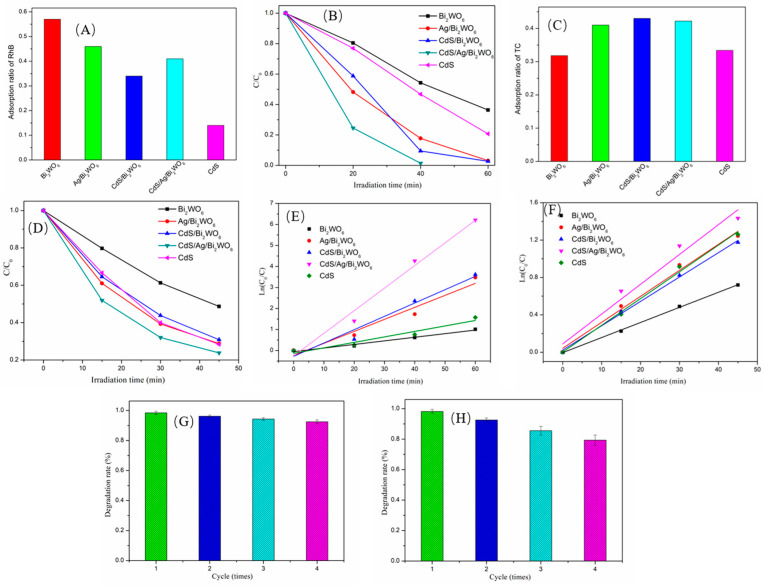
Adsorption ratio of RhB (**A**) and TC (**C**) in the dark; photodegradation of RhB (**B**) and TC (**D**) with different photocatalysts under visible light irradiation (λ ≥ 420 nm); the pseudo-first-order reaction kinetics of the RhB (**E**) and TC (**F**) degradation over different photocatalysts; cyclic photodegradation of RhB by CdS/Ag/Bi_2_WO_6_ photocatalyst with (**G**) and without (**H**) the regeneration procedure.

The photocatalytic performance of pure CdS, Bi_2_WO_6_, Ag/Bi_2_WO_6_, CdS/Bi_2_WO_6_, and CdS/Ag/Bi_2_WO_6_ was assessed via monitoring the photodegradation of RhB under visible light illumination (λ ≥ 420 nm). All catalysts were dispersed in RhB solution and then magnetically stirred in a dark environment for 60 min to attain an adsorption–desorption equilibrium. The adsorption capacity of all photocatalysts for RhB is shown in Figure 5A. The results indicated that the pure Bi_2_WO_6_ photocatalyst exhibits the highest adsorption capacity among all the photocatalysts, while the pure CdS photocatalyst has the lowest adsorption capacity. Additionally, Ag/Bi_2_WO_6_, CdS/Bi_2_WO_6_, and CdS/Ag/Bi_2_WO_6_ samples demonstrate similar capacities for RhB adsorption. From Figure 5B, it was found that the RhB degradation efficiencies over the Bi_2_WO_6_, CdS, Ag/Bi_2_WO_6_, CdS/Bi_2_WO_6_, and CdS/Ag/Bi_2_WO_6_ under visible light illumination for 40 min are about 45.8%, 53.2%, 82.2%, 90.5%, and 98.6%, respectively. Obviously, of all the as-prepared photocatalysts, Z-scheme CdS/Ag/Bi_2_WO_6_ heterojunction exhibited the greatest photocatalytic degradation effect, which was 2.15 and 1.85 times higher than the pure Bi_2_WO_6_ and CdS, respectively. The superior photocatalytic activity of CdS/Ag/Bi_2_WO_6_ could be because this Z-scheme ternary system has a stronger absorption capacity for visible light and excellent separation and transmission of photoinduced carriers compared to other photocatalysts.

In addition to RhB, other colorless pollutants, such as Tetracycline (TC), were also selected to estimate the photocatalytic efficiency of Bi_2_WO_6_, CdS, Ag/Bi_2_WO_6_, CdS/Bi_2_WO_6_, and CdS/Ag/Bi_2_WO_6_ under visible-light illumination (λ ≥ 420 nm). Before irradiation, the adsorption capacities of Bi_2_WO_6_, CdS, Ag/Bi_2_WO_6_, CdS/Bi_2_WO_6_, and CdS/Ag/Bi_2_WO_6_ were also subjected to 60 min in a dark environment. As depicted in Figure 5C, the adsorption ratios of Bi_2_WO_6_, CdS, Ag/Bi_2_WO_6_, CdS/Bi_2_WO_6_, and CdS/Ag/Bi_2_WO_6_ are approximately 0.318, 0.330, 0.410, 0.430, and 0.422, respectively. Unlike the adsorption of RhB, Ag/Bi_2_WO_6_, CdS/Bi_2_WO_6_, and CdS/Ag/Bi_2_WO_6_ have a higher adsorption capacity for TC. From Figure 5D, after the adsorption–desorption equilibrium, it can be discovered that the Z-scheme CdS/Ag/Bi_2_WO_6_ heterojunction presented the optimal photocatalytic activity, with a TC photodegradation efficiency of about 78% after 45 min of visible light illumination.

The kinetic behavior of the Bi_2_WO_6_, CdS, Ag/Bi_2_WO_6_, CdS/Bi_2_WO_6_, and CdS/Ag/Bi_2_WO_6_ photocatalysts in the RhB and TC degradation processes is displayed in Figure 5E and Figure 5F, respectively. Moreover, the pseudo-first-order equation was employed to fit the kinetic process. The Ln(C/C_0_) ∼ reaction time (t) curves exhibited linear variations, indicating that the photocatalytic degradation data of RhB and TC followed the first-level reaction kinetics law. The rate constants (RhB) of Bi_2_WO_6_, CdS, Ag/Bi_2_WO_6_, CdS/Bi_2_WO_6_, and CdS/Ag/Bi_2_WO_6_ were calculated as 0.0171, 0.0261, 0.0571, 0.0634, and 0.1075 min^−1^, respectively. Similarly, the rate constants (TC) of the Bi_2_WO_6_, CdS, Ag/Bi_2_WO_6_, CdS/Bi_2_WO_6_, and CdS/Ag/Bi_2_WO_6_ were determined as 0.0161, 0.0286, 0.0278, 0.0261, and 0.0319 min^−1^, respectively. It was evident that, compared with single- and two-component photocatalysts, the reaction rate of CdS/Ag/Bi_2_WO_6_ heterojunction has been significantly improved.

From the perspective of future practical applications, the repeatability and stability of the catalysts play a crucial role. Therefore, to evaluate the repeatability and stability of the CdS/Ag/Bi_2_WO_6_, recycling experiments were conducted using the heterojunction photocatalyst for RhB photodegradation (Figure 5G). The recovery process of the photocatalyst is as follows: after each degradation cycle, collect the photocatalyst powder, wash it three times with distilled water and ethanol, dry it, and proceed with subsequent photocatalysis. In the cyclic experiment, the error bars were derived from three batches of CdS/Ag/Bi_2_WO_6_ samples, representing the standard deviation. It can be observed that CdS/Ag/Bi_2_WO_6_ maintains high degradation efficiency after four cycles. As a comparison, in the cyclic experiment, no regeneration treatment was applied to directly use the collected photocatalyst powder. The results are shown in Figure 5H. After four cyclic experiments, there is a slight decrease in photocatalytic degradation efficiency compared to previous regeneration procedures. This may be attributed to pollutants adsorption on its surface after the experiment, which reduces active site availability and performance deterioration. This finding indicated remarkable stability of the Z-scheme CdS/Ag/Bi_2_WO_6_ heterojunction. 

### 3.5. Photocatalytic Mechanism of CdS/Ag/Bi_2_WO_6_ Heterojunction Photocatalyst

**Figure 6:**
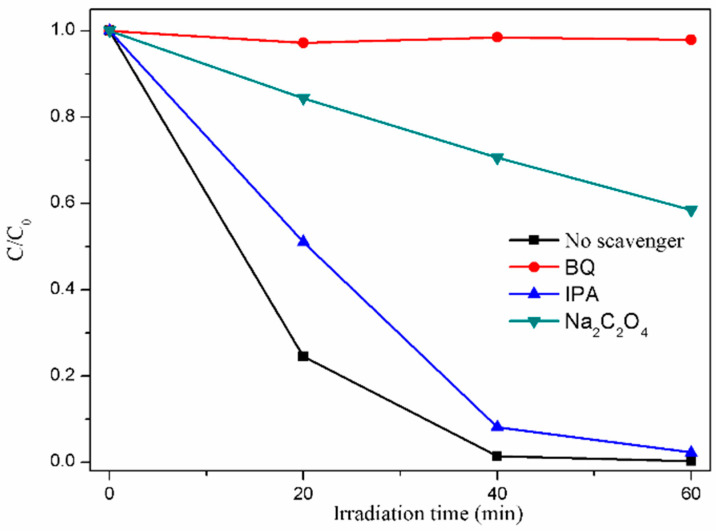
The species trapping experiments of CdS/Ag/Bi_2_WO_6_ under visible light irradiation.

Trapping experiments were performed to identify the key reactive radicals and further understand the probable catalytic mechanism. In the CdS/Ag/Bi_2_WO_6_ photocatalytic reaction system, isopropanol (IPA, 10 mM), sodium oxalate (Na_2_C_2_O_4_, 10 mM), and benzoquinone (BQ, 1 mM) were used as scavengers of hydroxyl radicals (^•^OH), holes (h^+^), and superoxide ions (^•^O_2_^−^), respectively. As displayed in Figure 6, the degradation efficiency of CdS/Ag/Bi_2_WO_6_ was slightly inhibited when IPA was added, implying that ^•^OH may not play a main role in the photodegradation of RhB. After adding BQ or Na_2_C_2_O_4_, the degradation performance of the CdS/Ag/Bi_2_WO_6_ photocatalyst was dramatically inhibited, which confirmed that ^•^O_2_^−^ and h^+^ were the key active species in the CdS/Ag/Bi_2_WO_6_ photocatalytic reaction.

**Figure 7:**
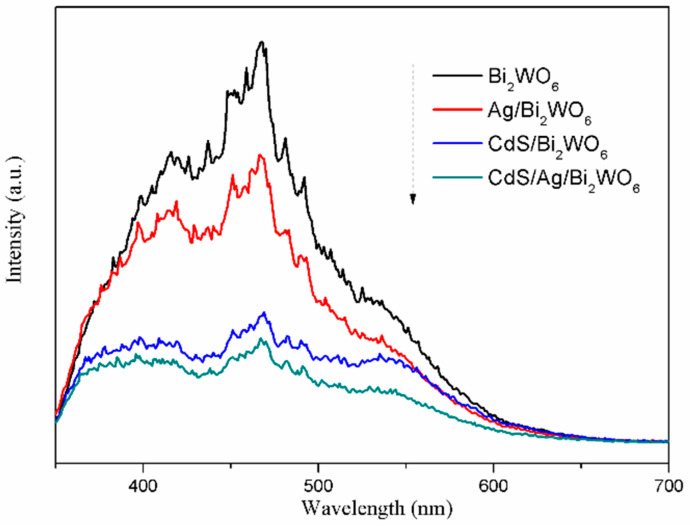
Photoluminescence spectra of Bi_2_WO_6_, Ag/Bi_2_WO_6_, CdS/Bi_2_WO_6_, and CdS/Ag/Bi_2_WO_6_.

The recombination efficiency of photoinduced electron-hole pairs was analyzed using photoluminescence (PL) spectroscopy. The weaker PL intensity typically indicates a lower possibility of photoinduced electron-hole recombination [52]. From Figure 7, the PL spectra of pure Bi_2_WO_6_, Ag/Bi_2_WO_6_, CdS/Bi_2_WO_6_, and CdS/Ag/Bi_2_WO_6_ are in the range of 350–700 nm, and all intense emission peaks are at about 468 nm. It is observed that the order of the emission intensity of the catalysts is: Bi_2_WO_6_ > Ag/Bi_2_WO_6_ > CdS/Bi_2_WO_6_ > CdS/Ag/Bi_2_WO_6_. The results confirmed that the CdS/Ag/Bi_2_WO_6_ heterojunctions possess the strongest separation efficiency of photoinduced charge carriers, suggesting they have superior photocatalytic performance.

**Figure 8:**
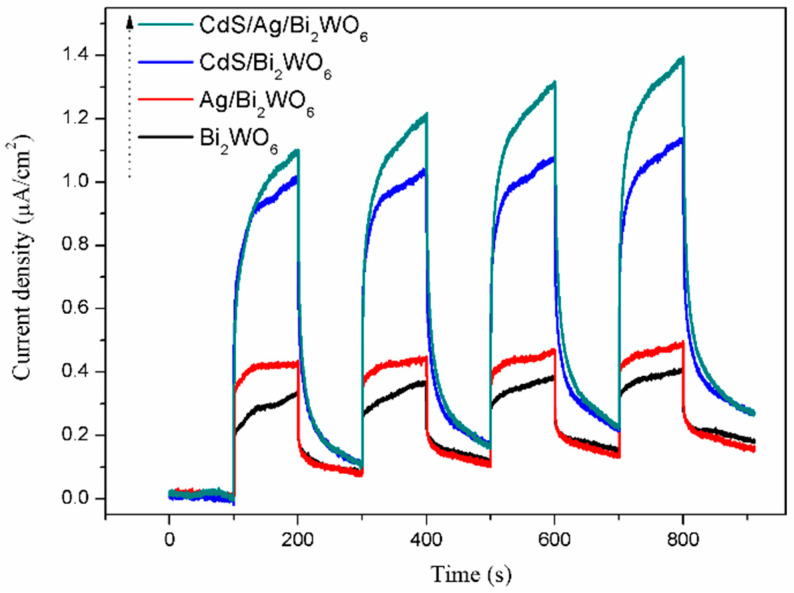
Photocurent responses of pure Bi_2_WO_6_, Ag/Bi_2_WO_6_, CdS/Bi_2_WO_6_, and CdS/Ag/Bi_2_WO_6_.

To further recognize the transmission and separation of photogenerated charge in CdS/Ag/Bi_2_WO_6_, the photocurrent response measurement is also employed under visible light irradiation. The higher photocurrent intensity means that the transmission efficiency of photogenerated carriers is higher, which results in outstanding photocatalytic activity [53]. Figure 8 shows the regular photocurrent responses of pure Bi_2_WO_6_, Ag/Bi_2_WO_6_, CdS/Bi_2_WO_6_, and CdS/Ag/Bi_2_WO_6_ in the dark and light. The photocurrent density increases in the order Bi_2_WO_6_, Ag/Bi_2_WO_6_, CdS/Bi_2_WO_6_, and CdS/Ag/Bi_2_WO_6_, which corresponds with their PL and photocatalytic properties. Therefore, given the above results, the as-prepared CdS/Ag/Bi_2_WO_6_ ternary heterojunction system can considerably enhance photogenerated charge transfer and separation efficiency, thereby improving photocatalytic performance. 

To systematically explore the photocatalytic mechanism, it is necessary to calculate the position of the conduction band (CB) and valence band (VB) using the following equation:

(1) $${\;E}_{\mathrm{VB}}=\chi -E_{e}+0.5E_{g}$$

(2) $${\;E}_{\mathrm{CB}}=E_{\mathrm{VB}}-E_{g}$$

*χ* value is the Mulliken electronegativity of Bi_2_WO_6_ (6.39 eV [54]) and CdS (5.18 eV [55]). The band gap values of Bi_2_WO_6_ and CdS were 2.81 and 2.17 eV, respectively. The calculated E_VB_ values of Bi_2_WO_6_ and CdS were 3.29 and 1.76 eV, respectively, and their corresponding E_CB_ values were 0.48 and −0.41 eV. With the determination of the CB and VB values of Bi_2_WO_6_ and CdS, the transport itinerary of photoinduced electron−hole pairs gradually becomes clear.

According to the charge carrier transfer mode of a typical type-II heterostructure, the photogenerated electrons on the CB of CdS will migrate to the CB of Bi_2_WO_6_. Because the conduction band of potential Bi_2_WO_6_ is more positive than the *E*(O_2_/^•^O_2_^−^) (−0.33 eV) [45], electrons on its CB cannot reduce O_2_ into ^•^O_2_^−^. However, from the results of trapping experiments, ^•^O_2_^−^ was the key active species in the CdS/Ag/Bi_2_WO_6_ photocatalytic reaction system. This means that the CdS/Ag/Bi_2_WO_6_ system has a different photoinduced electron and hole transport itinerary from the Bi_2_WO_6_/CdS system, which may be derived from the formation of the Z-scheme system with Ag-bridge as an efficient charge transfer medium. 

**Figure 9:**
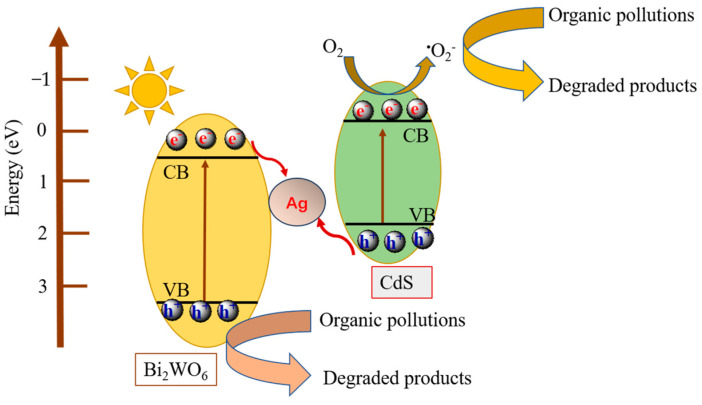
Schematic diagram of the proposed photogenerated charge separation and transmission over the CdS/Ag/Bi_2_WO_6_ heterojunctions under visible light irradiation.

Based on the analysis of the above result, a plausible mechanism for illustrating the transport path of photogenerated electron−hole pairs over the CdS/Ag/Bi_2_WO_6_ heterojunctions was proposed, as presented schematically in Figure 9. Upon exposure of the CdS/Ag/Bi_2_WO_6_ photocatalyst to visible light, both Bi_2_WO_6_ and CdS can be excited and then generate photogenerated electrons and holes. Because the CB position of Bi_2_WO_6_ is more negative than the Fermi level of silver nanoparticles, electrons on its CB will be injected into the silver nanoparticles through the Schottky barrier. Meanwhile, the holes on the VB of CdS will transfer to silver nanoparticles. Therefore, electrons generated from the CB of Bi_2_WO_6_ and holes generated from the VB of CdS can directly annihilate through the Ag nanoparticle bridge. The strong reductive electrons on the CB of CdS can react with dissolved oxygen molecules to form the active species ^•^O_2_^−^, which can oxidize organic contaminants into decomposed products. And the holes on the VB of Bi_2_WO_6_ oxidize organic contaminants directly. 

The Z-scheme with Ag-bridge in the CdS/Ag/Bi_2_WO_6_ ternary system not only promotes the spatial isolation of the photoinduced electron−hole pairs but also can maintain powerful redox capability, thus significantly boosting the quantum yield and photocatalytic activity.

## 4. Conclusions

In summary, the CdS/Ag/Bi_2_WO_6_ Z-scheme heterojunction photocatalysts were successfully synthesized by hydrothermal, photoreduction, and precipitation methods. Compared with single- and two-component systems such as CdS, Bi_2_WO_6_, Ag/Bi_2_WO_6_, and CdS/Bi_2_WO_6_ samples, the CdS/Ag/Bi_2_WO_6_ Z-scheme heterojunction exhibited remarkably boosted photocatalytic performance for the degradation of RhB and TC under visible light irradiation (λ ≥ 420 nm). The plausible photocatalytic mechanism was raised to explain the superior photocatalytic performance based on DRS and PL analysis, photocurrent responses, band edge positions, and the active species trapping experiment. In the CdS/Ag/Bi_2_WO_6_ Z-scheme heterojunctions system, the introduced Ag nanoparticles can be used as a bridge for the transportation of photogenerated charge carriers between CdS and Bi_2_WO_6_, thus accelerating photogenerated charge carrier separation and enhancing redox capacity. This work provided an effective method for the design and construction of extremely efficient photocatalysts based on semiconductor/noble-metal/semiconductor Z-scheme heterojunction composites.

## Data Availability

The data presented in the study are available from the corresponding author.
